# Supplementary material for: Inhibition of Notch pathway arrests PTEN-deficient advanced prostate cancer by triggering p27-driven cellular senescence
Source: Nat Commun. 2016 Dec 12;7:13719. doi: 10.1038/ncomms13719 (PMC5159884; doi:10.1038/ncomms13719)
Supplement: Supplementary Information — Supplementary Figures and Supplementary Tables [file ncomms13719-s1.pdf]

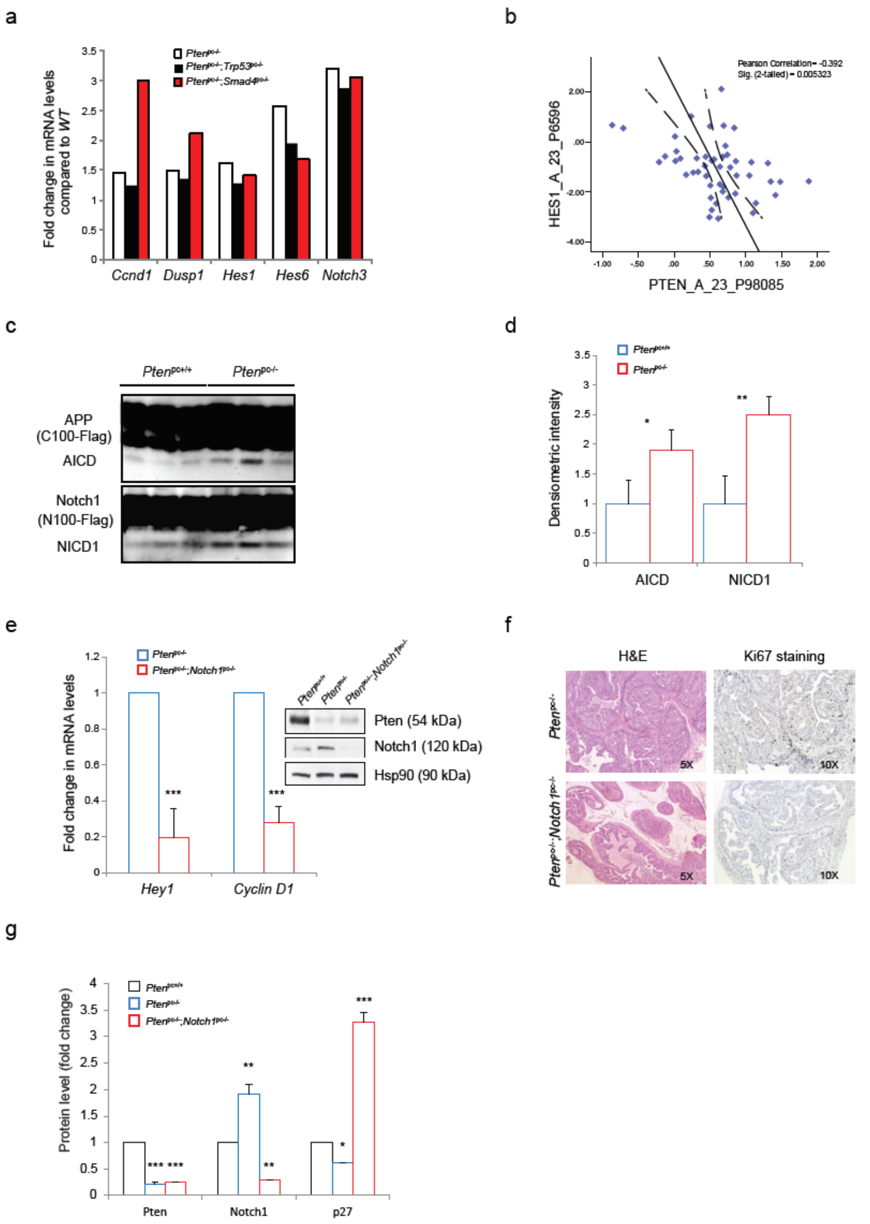

Supplementary Figure 1 | Notch pathway activation in different mouse models and in human prostate cancers. (**a**) Fold change in gene expression profile of Notch signaling pathway target genes in *Pten*$^{pc-/-}$, *Pten*$^{pc-/-}$; *Trp53*$^{pc-/-}$ and *Pten*$^{pc-/-}$; *Smad4*$^{pc-/-}$compared to *WT*. (**b**) Graph showing the inverse correlation between the mRNA levels of *PTEN* and *HES1* in a human prostate cancer dataset. (**c**) Enzymatic *in vitro* assay showing the elevated activity of the γ-secretase complex in *Pten*$^{pc-/-}$ prostate tumors (n=3). (**d**) Quantification of **c**. (**e**) Fold change in mRNA levels of *Hey1* and *Cyclin D1* target genes of Notch pathway in *Pten*$^{pc-/-}$;*Notch1*$^{pc-/-}$ tumors compared to *Pten*$^{pc-/-}$ tumors. WB showing the protein level of Pten and Notch1 in *Pten*$^{pc+/+}$, *Pten*$^{pc-/-}$ and *Pten*$^{pc-/-}$;*Notch1*$^{pc-/-}$ prostate tumors. (**f**) H&E and Ki67 staining in staining of APs derived from 12 weeks old *Pten*$^{pc-/-}$ and *Pten*$^{pc-/-}$;*Notch1*$^{pc-/-}$ tumors (n=3). Magnification 5x and 10x respectively. (**g**) Quantification of WB from Fig. **1h**. Values are expressed as mean ± SEM. *p<0.05; **p<0.01; ***p<0.001 by Student's t-test.

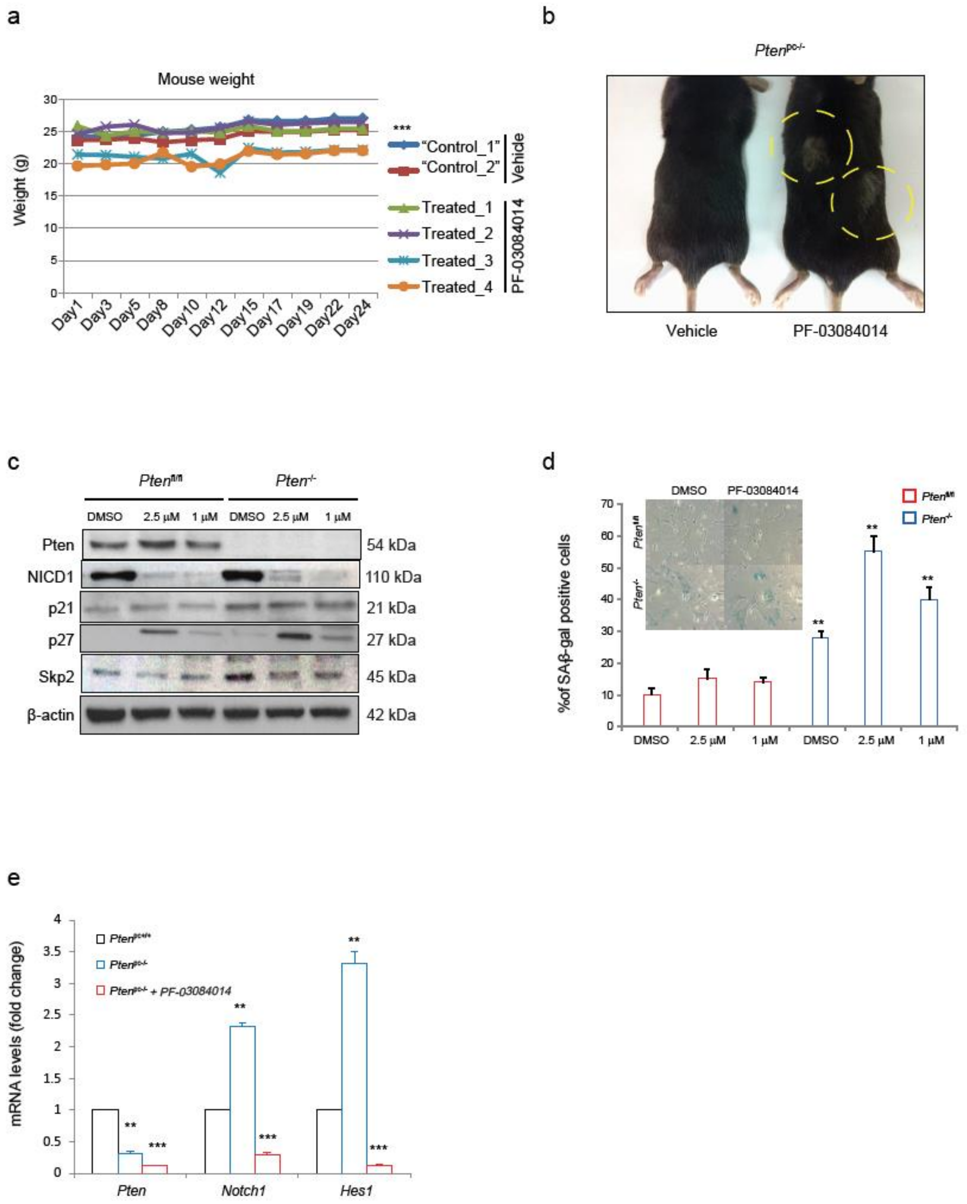

Supplementary Figure 2 | *Pten*$^{pc-/-}$ mice well tolerate PF-03084014 treatment, efficacy of PF-03084014 *Pten*-null MEFs and activated Notch1 transcribes itself. (**a**) Graph representing the weight of *Pten*$^{pc-/-}$ mice treated with either vehicle or PF-03084014. (**b**) Image showing (yellow circles) the spotted white hair phenotype observed in *Pten*$^{pc-/-}$ mice treated with PF-03084014. (**c**) WB showing the effect of PF-03084014 on the protein levels of NICD1, p21, p27 and the Notch1 target gene Skp2 in *Pten*$^{fl/fl}$ and *Pten*$^{-/-}$ MEFs. (**d**) Graph showing the percentage of senescent cells in *Pten*$^{fl/fl}$ and *Pten*$^{-/-}$ MEFs after treatment with PF-03084014. MEFs were treated daily for 4 days at the indicated concentration. (**e**) Fold change in mRNA levels of *Pten*, *Notch1* and *Hes1* in WT, *Pten*$^{pc-/-}$ and *Pten*$^{pc-/-}$ treated with PF-03084014. Values are expressed as mean ± SEM. $^{**}p<0.01$; $^{***}p<0.001$ by Student's t-test.

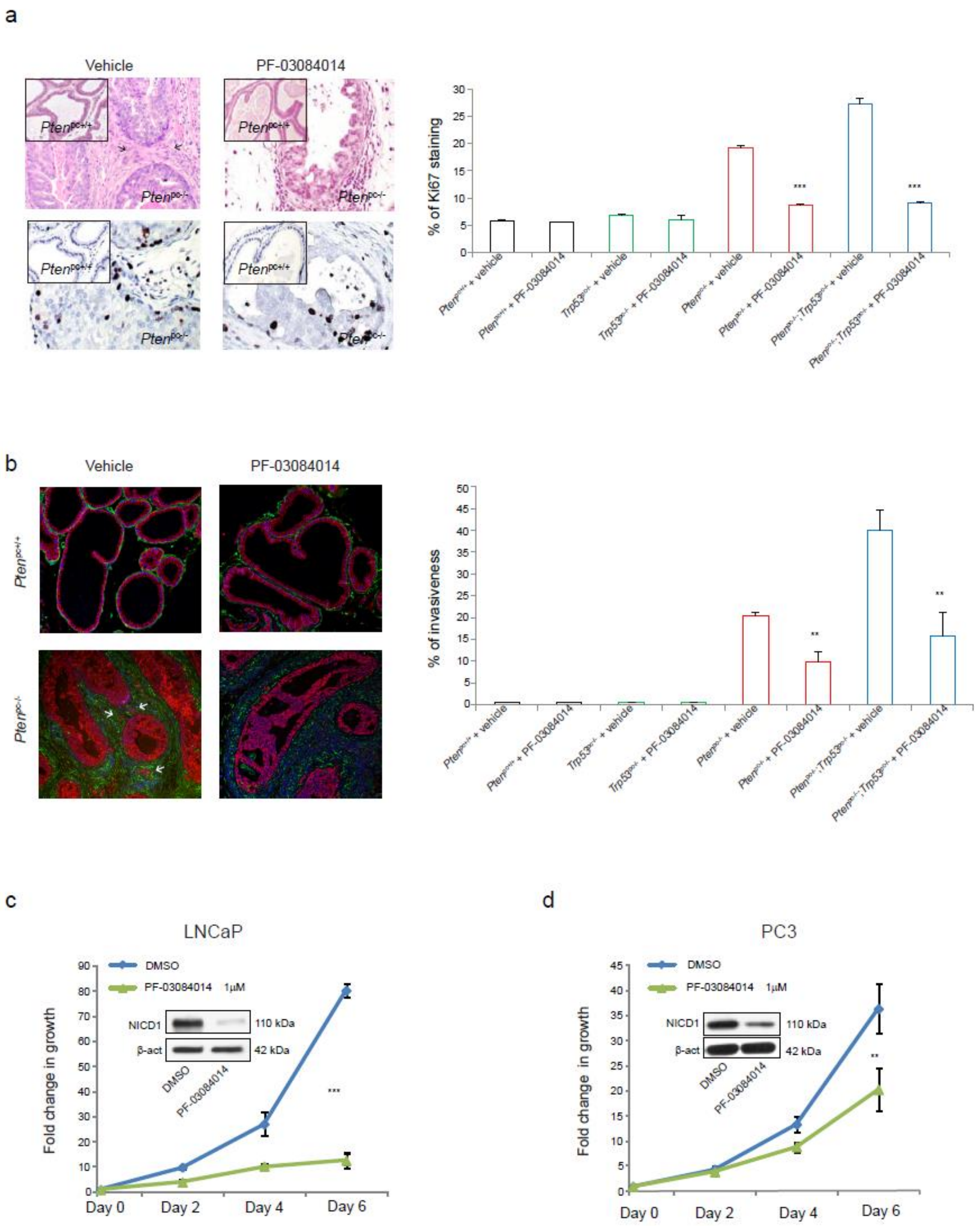

Supplementary Figure 3 | Anti-proliferative effect of PF-03084014 in mouse tumours and human PCa cells. (**a**) Representative H&E and Ki67 staining images of *Pten*$^{pc+/+}$ (inset) and *Pten*$^{pc-/-}$ tumours treated with vehicle and PF-03084014 (left panel). Arrows in black indicating stroma-infiltrating epithelial-tumor cells. Quantification of Ki67 in mice of indicated genotypes (right panel). Magnification 20x and 40x respectively (n=5). (**b**) Representative IF images for Vimentin/E-Cadherin staining in *Pten*$^{pc+/+}$ and *Pten*$^{pc-/-}$ tumours treated with vehicle and PF-03084014. White arrows indicate stromal-invasion of tumor cells. Right panel quantification of percentage of tumours with invasive prostate glands in mice of indicated genotypes. Magnification 10x (n=5). (**c**) Growth curve of LNCaP cells treated either with DMSO or PF-03084014. (**d**) Growth curve of PC3 cells treated either with DMSO or PF-03084014. Values are expressed as mean ± SEM. $**p<0.01$; $***p<0.001$ by Student's t-test.

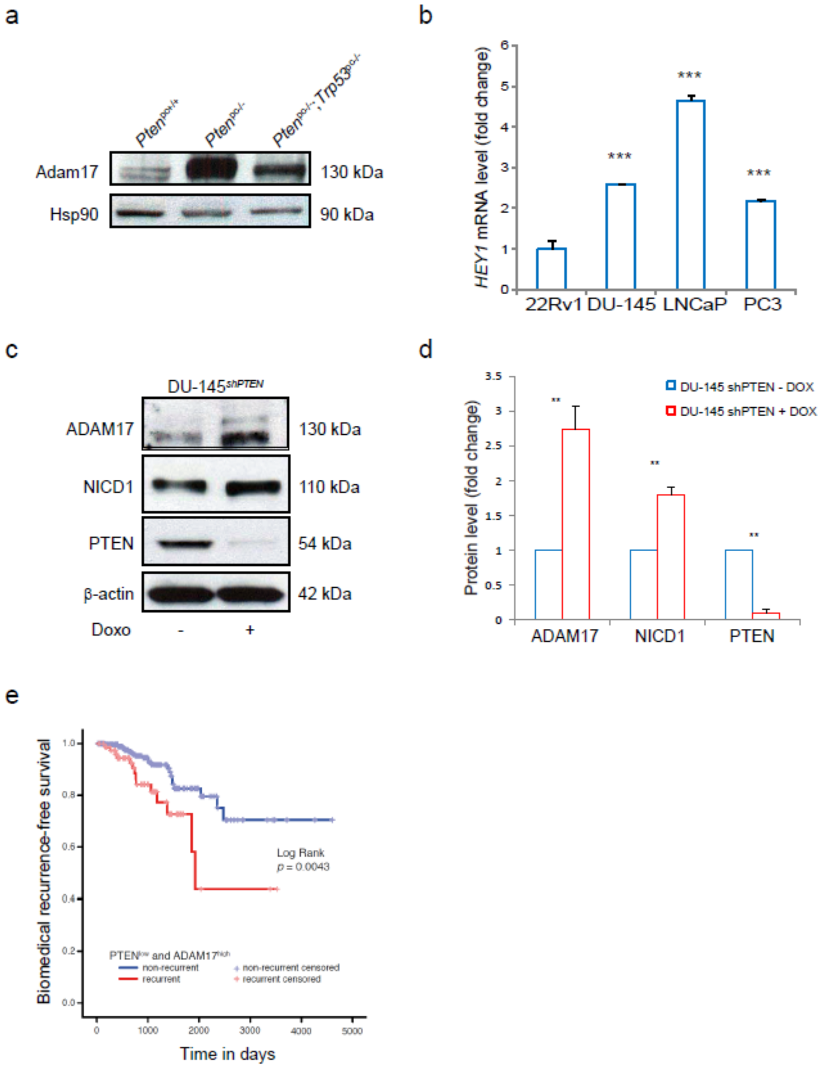

Supplementary Figure 4 | ADAM17 expression. (**a**) WB showing the protein levels of Adam17 in *Pten*$^{pc+/+}$ wt prostate, *Pten*$^{pc-/-}$ and *Pten*$^{pc-/-}$*; Trp53*$^{pc-/-}$ prostate tumors. (**b**) HEY1 mRNA levels in different PCa cell lines. (**c**) WB showing the levels of ADAM17, NICD and PTEN in DU-145^{shPTEN} cell line treated with Doxycycline (Dox). (**d**) Quantification of **c**. (**e**) Kaplan-Meier curve showing that prostate cancer patients with low levels of *PTEN* and high levels of *ADAM17* have a worse prognosis. Values are expressed as mean ± SEM. **p<0.01; ***p<0.001 by Student's t-test.

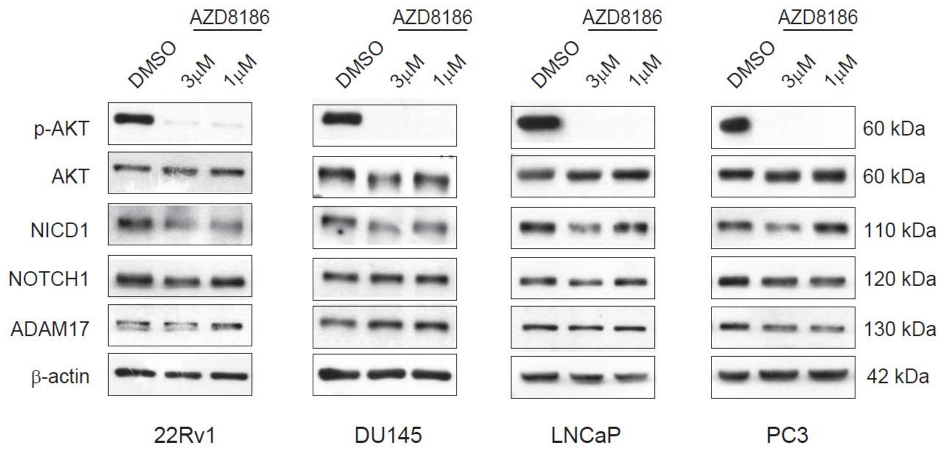

Supplementary Figure 5 | Activation of Notch signaling pathway in PTEN-deficient cells is PI3K/AKT-independent. WB analysis in human PCa cell lines 22Rv1, DU145, LNCaP and PC3 showing protein levels of p-AKT, total AKT, NICD1, NOTCH1 and ADAM17 upon treatment with DMSO and PI3K inhibitor, AZD8186.

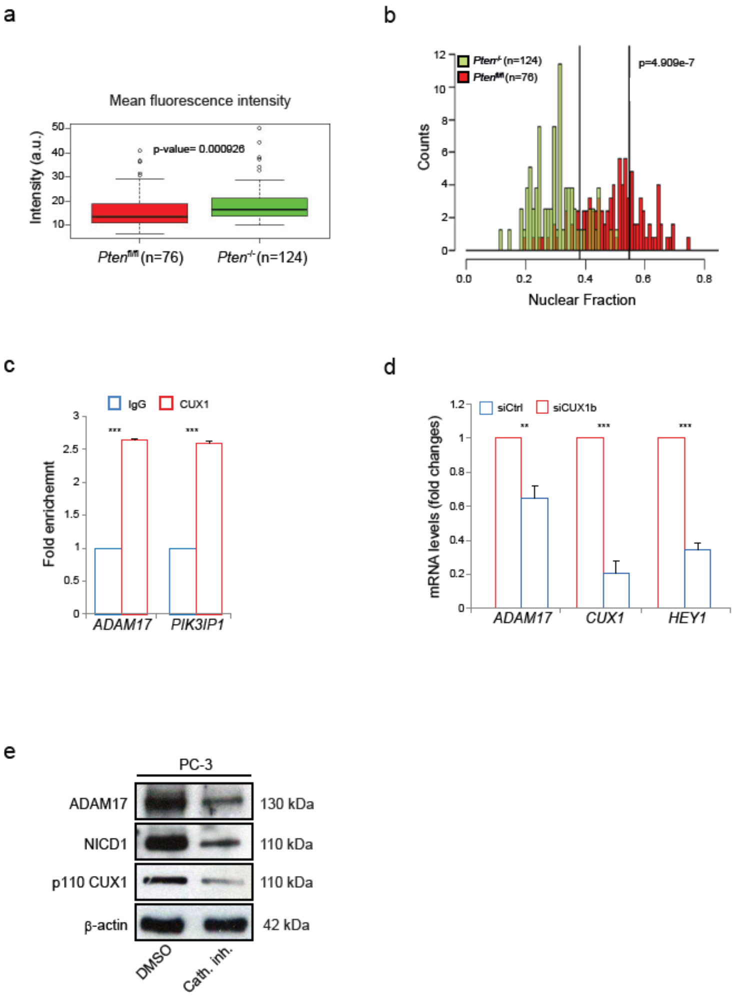

Supplementary Figure 6 | CUX1 expression, localization and ChIP in MEFs and DU-145 cells. (**a**) Cux1 mean fluorescence intensity in *Pten*$^{fl/fl}$ and *Pten*$^{-/-}$ MEFs. (**b**) Cux1 Nuclear fraction in *Pten*$^{fl/fl}$ and *Pten*$^{-/-}$ MEFs. (**c**) CUX1-ChIP in DU-145 cells. Graph showing the fold enrichment for *ADAM17* and *PIK3IP1*. (**d**) mRNA levels of *ADAM17* and *CUX1* in DU-145 cells transfected with *siCUX1*. (**e**) WB analysis showing the reduction in NICD1 and ADAM17 upon use of CathepsinL inhibitor in PC3 cells. Values are expressed as mean ± SEM. **$p<0.01$; ***$p<0.001$ by Student's t-test.

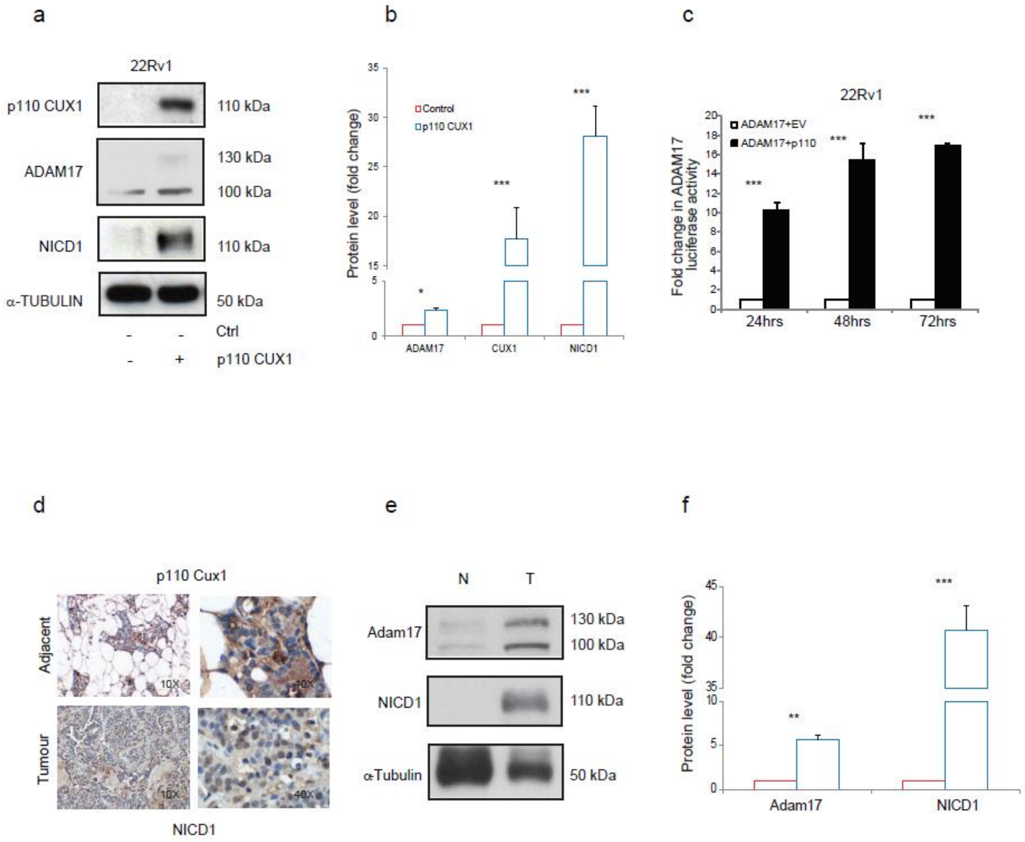

Supplementary Figure 7 | Overexpression of p110CUX1 increases ADAM17 levels and the activity of NOTCH. (**a**) WB analysis in 22Rv1 cells transfected with p110 CUX1 or empty vector. (**b**) Quantification of **a**. (**c**) ADAM17 luciferase activity in cells transfected as in **a** evaluated at different time points. (**d**) Immunohistochemistry for NICD1 in p110 Cux1 breast tumors at both not invasive and invasive stage. Magnification 10x and 40x. (**e**) WB analysis for Adam17 and NICD1 in p110 Cux1 breast cancer and normal mammary glands. (**f**) Quantification of **e**. Values are expressed as mean ± SEM. *p<0.05; **p<0.01; ***p<0.001 by Student's t-test.

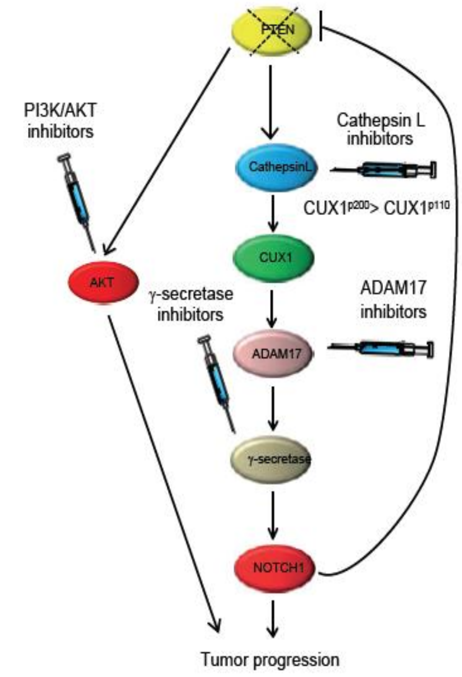

Supplementary Figure 8 | The Model. In prostate tumors with loss of PTEN, the high levels of CathepsinL promote the conversion of p200 CUX1 into its oncogenic isoform p110 CUX1, which in turn binds the promoter of ADAM17 and activates its transcription. ADAM17 then leads to the activation of Notch1 signaling which sustains tumor progression and may further reduce *PTEN* levels. This novel oncogenic network functions independently of PI3K/AKT pathway in the cells having loss of function of PTEN. This study may provide multiple entry points to design novel therapeutic intervention for PCa (e.g. CathepsinL inhibitors, ADAM17 inhibitors, γ-secretase inhibitors and AKT inhibitors).

Figure 1b

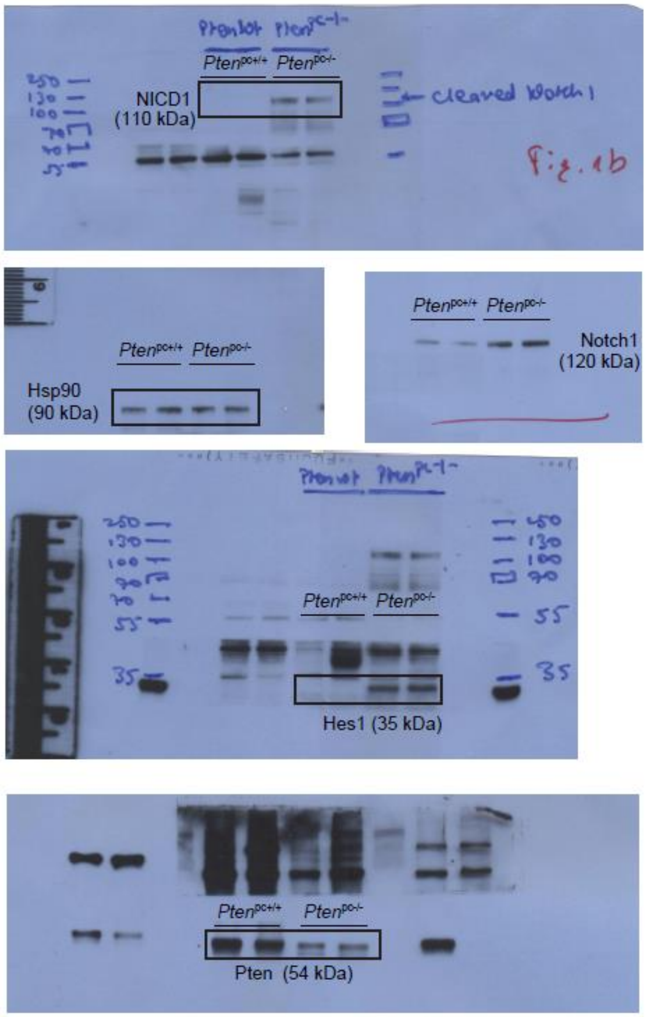

Figure 1h

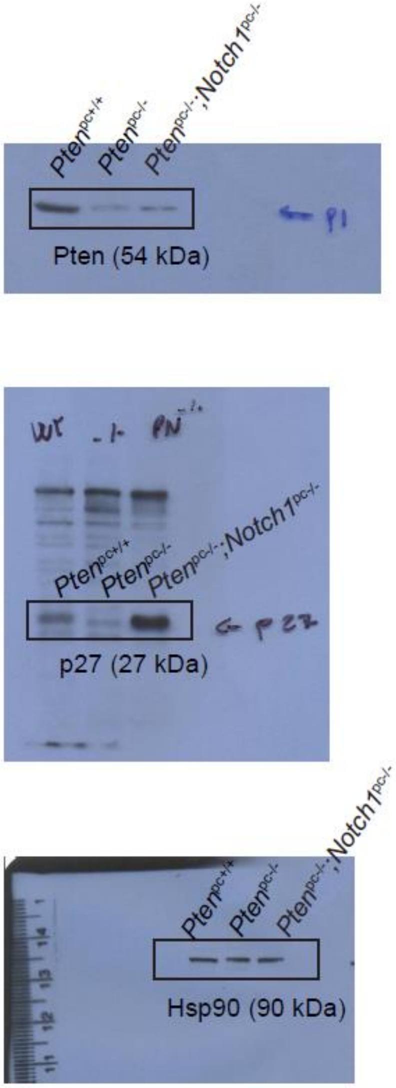

Supplementary Figure 9 | Images of western blots in full (Fig. 1 on this page)

Figure 2g

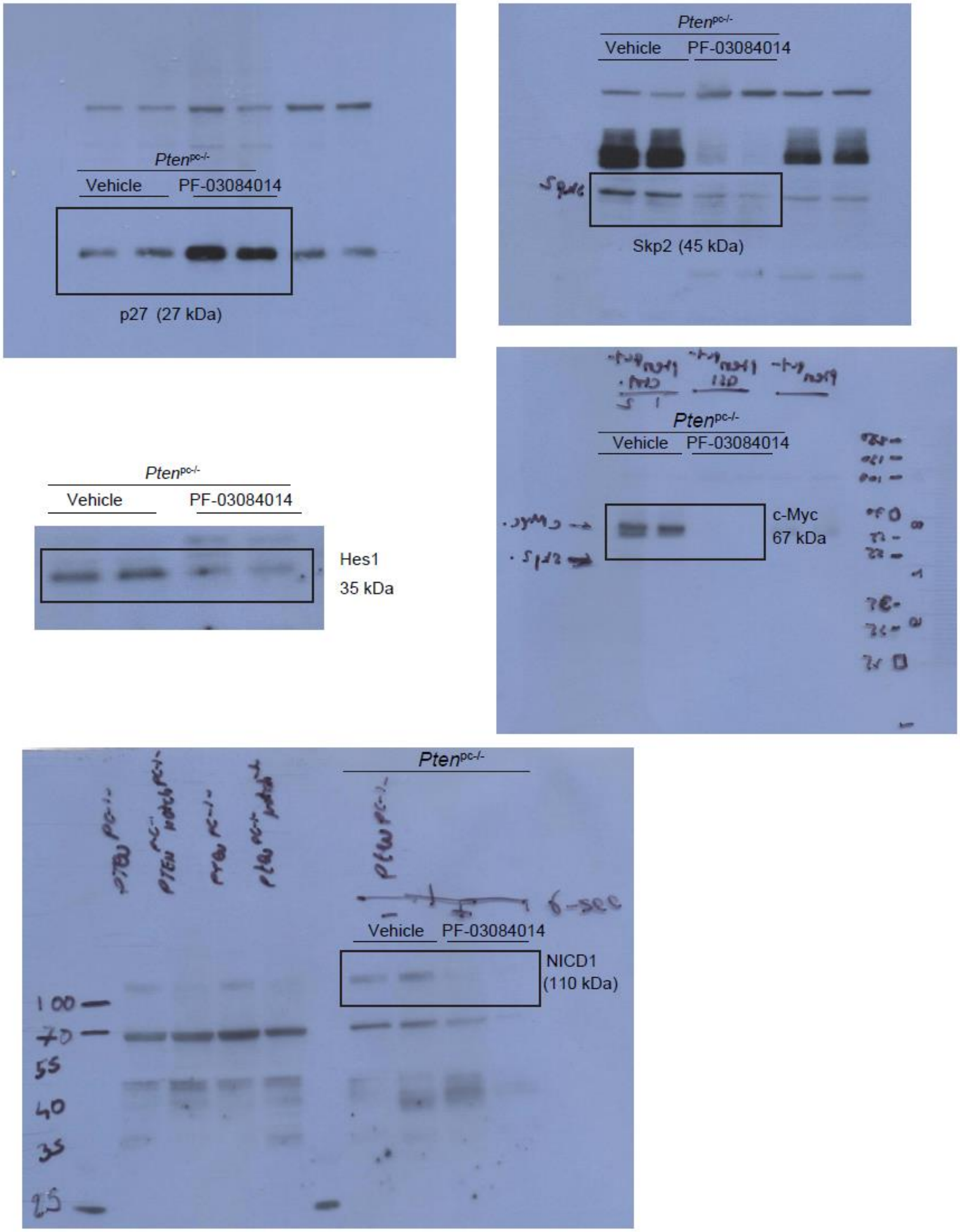

Supplementary Figure 9 (continued full scans of western blots from Fig. 2)

Figure 3b

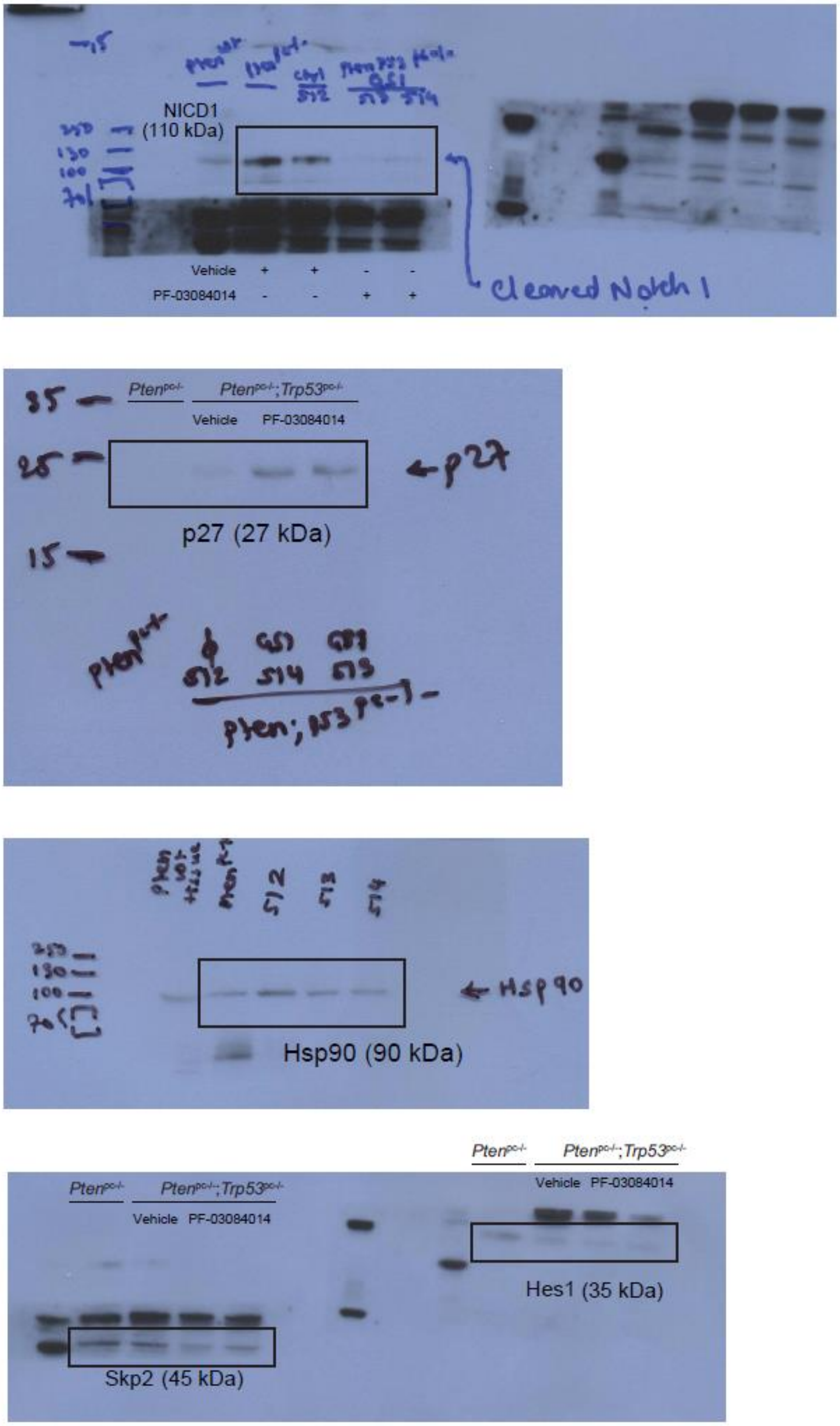

Supplementary Figure 9 (continued full scans of western blots from Fig. 3)

Figure 4d

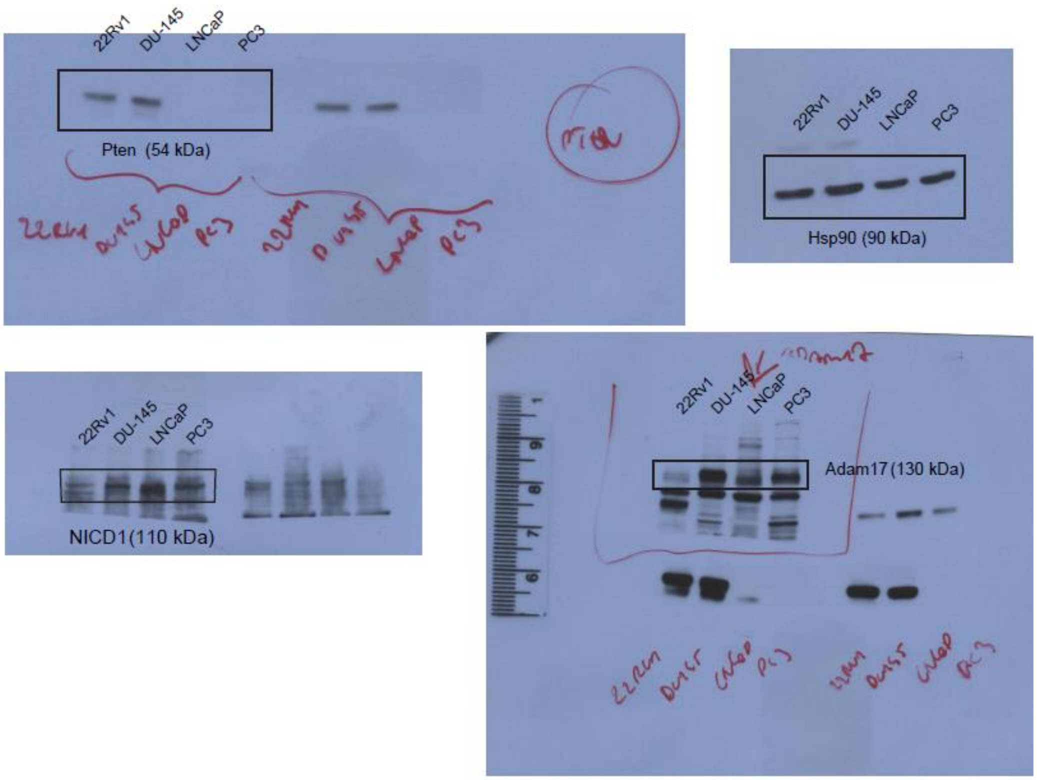

Figure 4j

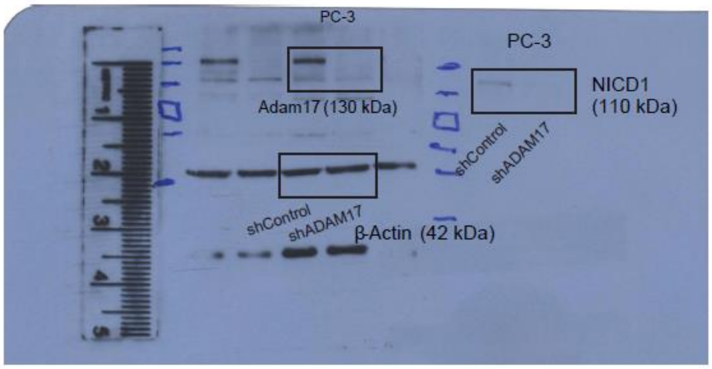

Supplementary Figure 9 (continued full scans of western blots from Fig. 4)

Figure 5a

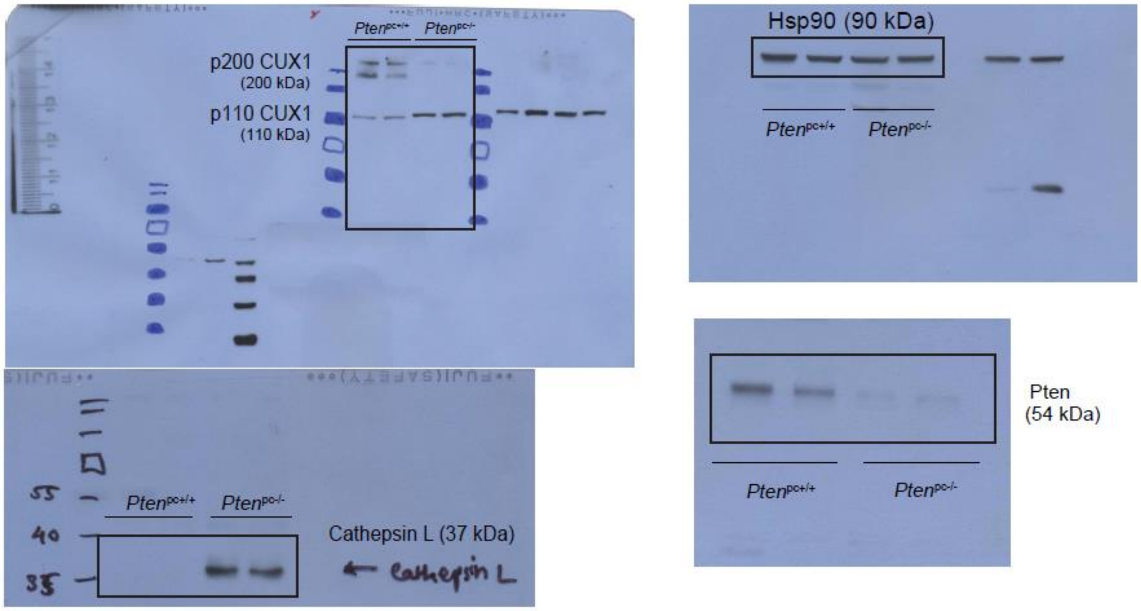

Figure 5f

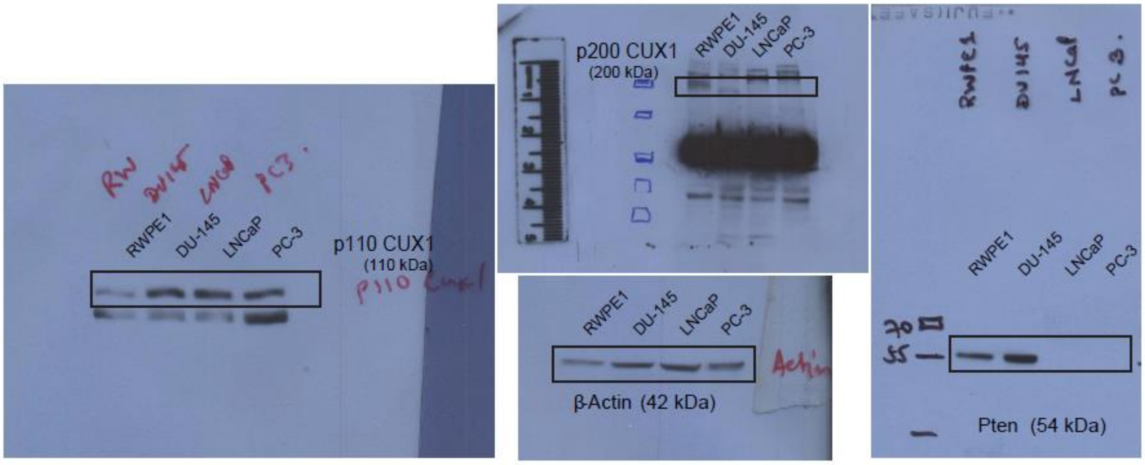

Supplementary Figure 9 (continued full scans of western blots from Fig. 5a,f)

Figure 5h

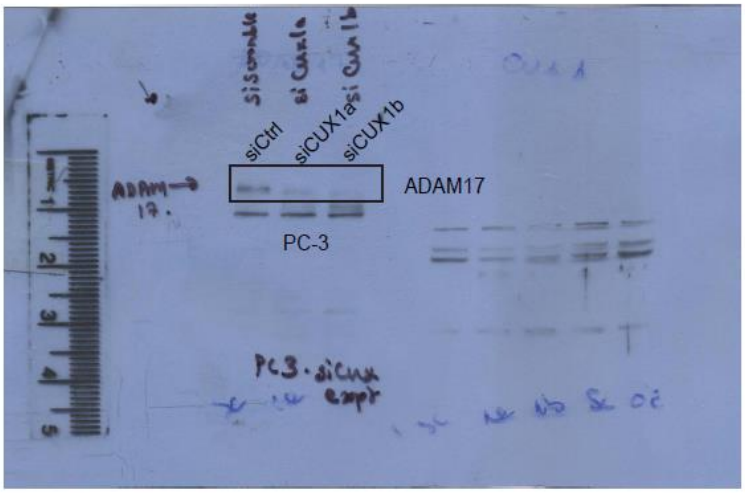

Figure 5c

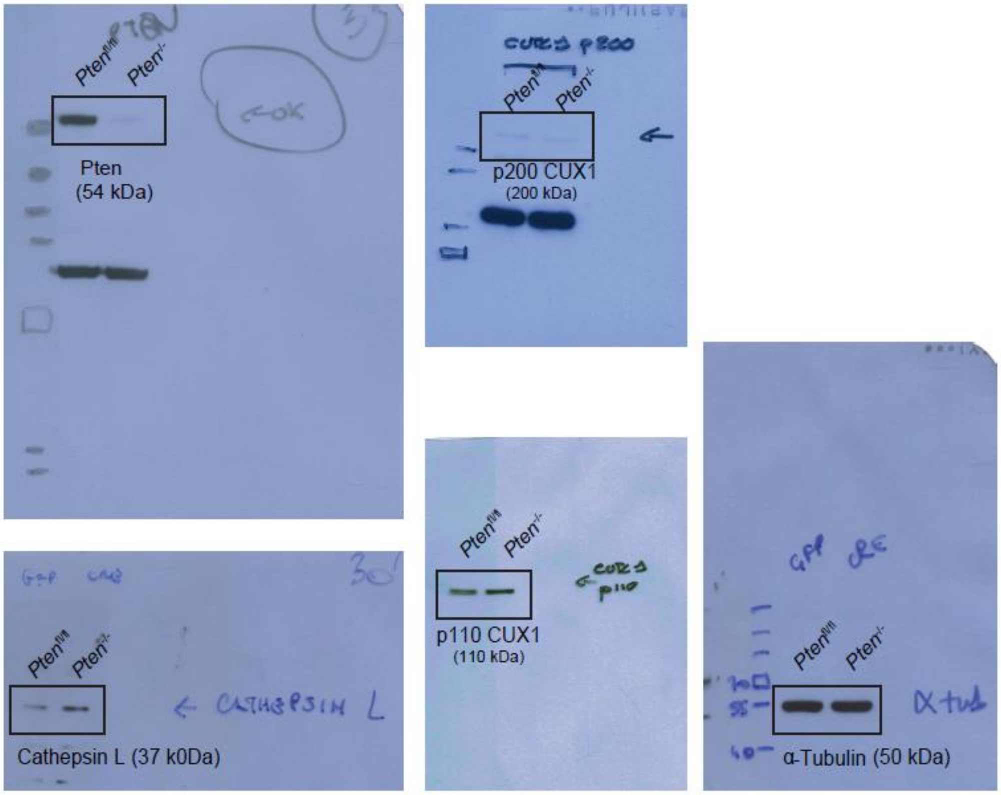

Supplementary Figure 9 (continued full scans of western blots from Fig. 5h,c)

ADAM17 promoter	
Mouse	Human
RORalpha2	HOXA3
POU6F1(c2)	CUTL1
CUTL1	ATF
RelA	IRF-1
RORalpha1	IRF-7A
C/EBP alpha	AML 1a
POU3F2	AP-1
POU3F2 (N.Oct-5a)	ATF
POU3F2 (N.Oct-5b)	Bach1
POU3F1	C/EBP alpha
AML 1a	CUTL1
AP-1	Elk-1
ATF-2	En-1
Bach1	Evi-1
C/EBP alpha	HNF-4alpha1
CUTL1	HOXA3
Chx10	HIF
E4BP4	IRF-1
Elk-1	IRF-7A
En-1	IK-1
Evi-1	MEF-2a
FAC1	Nrf-2a
FPXJ2	Nkx2-5
XJ2 (long isoform)	Nkx 6-1
FPXL1	Olf-1

Supplementary Table 1 | List of predicted transcription factors Top 25 transcription factors predicted to bind the promoter region of both mouse and human *ADAM17* gene based on SABiosciences' proprietary database (DECODE, DECipherment Of DNA Elements). Indicated by font color are other common transcription factors while highlighted is the transcription factor selected.

Vadnais DATASET

Hs578t cells	ADAM17	o.e. p110 CUX1^{up}
	shCUX1^{down}	p=0.000055
	HEY1	o.e. p110 CUX1^{up}
	shCUX1^{down}	p=0.000001

Supplementary Table 2 | ADAM17 regulation in Hs578T cells before and after overexpression and down regulation of p110 CUX1

Primer	Sequence 5’-3’
PIK3IP1Qf	GCTGGCATCATCTTGGGCTACT
PIK3IP1Qr	AGACAAGGGCAGAGTGATTCGC
CUX1Qf	TCCGTAGCATCCAAGGCAGACA
CUX1Qr	CTTCATCAGAGCCAGTCTCCGA
Cux18F	CCAAGAACGGCATTTGCCAGAG
Cux19R	CCTTTCTGGGTCAGTTTGCTCC
β-actin_H_ Fwd	TCCCTGGAGAAGAGCTACGA
β-actin_H_ Rev	AGGAAGGAAGGCTGGAAGAG
PTEN_H_Fwd	AGCCGTTCGGAGGATTATTCG
PTEN_H_Rev	CTTCTCCTCAGCAGCCAGAG
Adam17_m_Fwd	AGGACGTAATTGAGCGATTTTGG
Adam17_m_Rev	TGTTATCTGCCAGAAACTTCCC
Pten_m_Fwd	TGGATTCGACTTAGACTTGACCT
Pten_m_Rev	GCGGTGTCATAATGTCTCT
18s_m_Fwd	AAGTCCCTGCCCTTTGTACACA
18s_m_Rev	GATCCGAGGGCCTCACTAAAC
ADAM17_H_Fwd	ACTCTGAGGACAGTTAACCAAACC
ADAM17_H_Rev	AGTAAAAGGAGCCAATACCACAAG
HEY1_H_Fwd	GAAGTTGCGCGTTATCTGAGC
HEY1_H_Rev	ATGCGAAACCAGTCGAACTCG

Supplementary Table 3 | List of primers used for RT-PCR
